# A Separator with Double Coatings of Li_4_Ti_5_O_12_ and Conductive Carbon for Li‐S Battery of Good Electrochemical Performance

**DOI:** 10.1002/advs.202301386

**Published:** 2023-05-18

**Authors:** Shuang Xia, Jie Song, Qi Zhou, Lili Liu, Jilei Ye, Tao Wang, Yuhui Chen, Yankai Liu, Yuping Wu, Teunis van Ree

**Affiliations:** ^1^ State Key Laboratory of Materials‐oriented Chemical Engineering School of Energy Science and Engineering Nanjing Tech University Nanjing Jiangsu 211816 China; ^2^ School of Energy and Environment South East University Nanjing Jiangsu 211189 China; ^3^ Hunan Bolt Power New Energy Co. Ltd., Dianjiangjun Industrial Park Louxing District Hunan Road Loudi 417000 China; ^4^ Department of Chemistry University of Venda Thohoyandou 0950 South Africa

**Keywords:** Li‐S batteries, lithium anode corrosion, lithium dendrites, modification, separator, shuttle effect

## Abstract

The market demand for energy pushes researchers to pay a lot of attention to Li‐S batteries. However, the ‘shuttle effect’, the corrosion of lithium anodes, and the formation of lithium dendrites make the poor cycling performances (especially under high current densities and high sulfur loading) of Li‐S batteries, which limit their commercial applications. Here, a separator is prepared and modified with Super P and LTO (abbreviation SPLTOPD) through a simple coating method. The LTO can improve the transport ability of Li^+^ cations, and the Super P can reduce the charge transfer resistance. The prepared SPLTOPD can effectively barrier the pass‐through of polysulfides, catalyze the reactions of polysulfides into S^2−^, and increase the ionic conductivity of the Li‐S batteries. The SPLTOPD can also prevent the aggregation of insulating sulfur species on the surface of the cathode. The assembled Li‐S batteries with the SPLTOPD can cycle 870 cycles at 5 C with the capacity attenuation of 0.066% per cycle. When the sulfur loading is up to 7.6 mg cm^−2^, the specific discharge capacity at 0.2 C can reach 839 mAh g^−1^, and the surface of lithium anode after 100 cycles does not show the existence lithium dendrites or a corrosion layer. This work provides an effective way for the preparation of commercial separators for Li‐S batteries.

## Graphical abstract

A multifunctional separator is prepared by coating the Super P and LTO onto DKJ‐14 separator for the Li‐S battery. The lithium anodes are uncorroded and dendritic free, the ‘shuttle effect’ is greatly depressed, and the charge transfer resistance is reduced, leading to high‐performance Li‐S batteries.

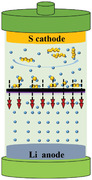

## 1 Introduction

With the development of electric vehicles, the market has higher requirements for energy storage devices.^[^ ^1^, ^2^ ^]^ Batteries with higher energy density and longer cycle life could meet the needs of the market.^[^ ^3^, ^4^ ^]^ Li‐S batteries have attracted extensive attention due to the high theoretical capacity of S (1675 mAh g^−1^) and energy density (2600 Wh kg^−1^). In addition, the advantages of sulfur, which has low cost, is easily available, and environmentally friendly, have also attracted wide attentions.^[^ ^5^, ^6^, ^7^, ^8^ ^]^ However, The ‘shuttle effect’ and lithium anode corrosion have always restricted the development of Li‐S batteries. During the charging and discharging process, the soluble lithium polysulfides (Li_2_S_x_, 2 < *x* ≤ 8) are formed and dissolved in the electrolyte, resulting in the loss of active sulfur and corrosion of lithium anodes, which leads to the sharp capacity decline and poor safety performance.^[^ ^9^, ^10^, ^11^ ^]^

Many efforts have been made to solve the above problems in Li‐S batteries. As for the cathodes, various materials and structures are designed to anchor the active sulfur. Different carbon materials (such as carbon nanotubes, biomass carbon, and porous carbon) are used as the hosting framework for sulfur cathodes.^[^ ^12^, ^13^, ^14^, ^15^, ^16^, ^17^ ^]^ Besides, inorganic compounds are often used to tailor the cathodes because of their excellent adsorption or catalysis on polysulfides, ^[^ ^18^, ^19^ ^]^ which improves the utilization of sulfur and accelerates the redox reaction kinetics. For the anodes, many measures are taken to prevent the corrosion of lithium anodes and the growth of lithium dendrites, among which the method of constructing artificial protective layers on lithium anodes is effective.^[^ ^20^, ^21^, ^22^, ^23^ ^]^ These mentioned strategies have greatly improved the electrochemical performance of Li‐S batteries, but the tailored preparation processes for positive and negative electrode structures often lead to an increase in time and cost, which restricts the further development of Li‐S batteries.

As an important part of the batteries, the separators play an important role in preventing short circuits and maintaining lithium ions. In recent years, it was found that simple modification of the separators can greatly improve the electrochemical performance of the batteries.^[^ ^24^, ^25^ ^]^ The idea of separator modification is to add functional modification layers on the separators to prevent the active sulfur from moving to the anode side. The modified material should have the function of accelerating redox reactions, forming a stable modification layer on the separators, and improving the ionic transport ability. In recent years, there are quite some works related to the modification on separators, and outstanding progresses have undoubtedly been achieved.^[^ ^26^, ^27^, ^28^, ^29^ ^]^ The simple, fast, and low‐cost coating method for preparing modified separators can greatly improve the electrochemical performance of Li‐S batteries.^[^ ^30^, ^31^ ^]^ However, the prepared Li‐S batteries are difficult to have good cycling performance under high current density and high sulfur loading, which are not conducive to the commercialization of Li‐S batteries. For the separators that can promote the commercialization of Li‐S batteries, it is supposed that they should have the advantages of cheap modified materials, simple preparation, and short preparation time. They can also simultaneously suppress the ‘shuttle effect’ and protect the anodes. In addition, the prepared Li‐S batteries have excellent cycling performance under high sulfur loading and high current density.

Here, we prepared a separator modified with Li_4_Ti_5_O_12_ (LTO) and a conductive carbon (Super P) (SPLTOPD) for Li‐S batteries, the Super P modified separators (SPD), and the LTO modified separators (LTOPD) were prepared for comparison, too. LTO has the advantages of low cost and good stability as commercial anode material for lithium ion batteries.^[^ ^32^, ^33^, ^34^, ^35^ ^]^ Furthermore, it can improve the Li^+^ transport ability. It can also form a stable additional layer on the separators to barrier the transport of polysulfides, which improves the utilization of active sulfur and accelerates the redox kinetics of S electrode.

In 2016, Yang et al. prepared a Li_4_Ti_5_O_12_/graphene coated separator for Li‐S batteries.^[^ ^36^ ^]^ The above work demonstrates the feasibility of using LTO nanospheres as modified materials for separators of Li‐S batteries, but the preparation of special structure LTO will increase costs. In our work, we found that commercial LTO together with PVDF in combination with Super P conductive carbon could be used as the modification material for separator of Li‐S batteries. It is found the Super P modified layers on the separators can improve the utilization of insulating active substances, buffer the shuttle of polysulfides, and provide a reaction site for polysulfides.^[^ ^37^, ^38^ ^]^ Super P as an additional layer on the LTO modified layer can further improve the barrier of the separators to polysulfides and reduce the charge transfer resistance of the batteries.^[^ ^39^ ^]^ The prepared Li‐S batteries show excellent cycling performance under high current density and high sulfur loading.

## 2 Results and Discussion

**Figure 1:**
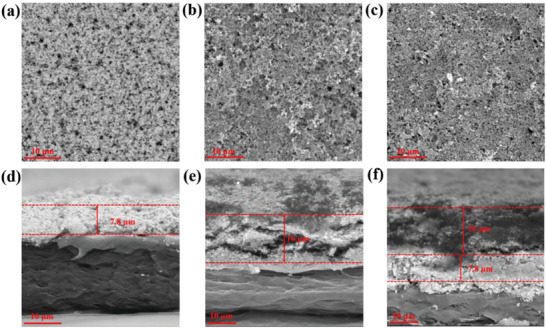
SEM of different separators. The surface of the a) LTOPD, b) SPD, c) SPLTOPD, and d–f) the corresponding section.

The LTO was tested by XRD (Figure S1, Supporting Information), and it corresponds to the peak of spinel lithium titanate (Li_4_Ti_5_O_12_, JCPDS No. 49–0207). The LTO powders were pressed into a pellet and assembled in stainless steel//stainless steel (SS//SS) cells for the EIS test, showing an ionic conductivity of 2.53 mS cm^−1^ at 25 °C (Figure S2, Supporting Information). The good transport ability of commercial LTO itself for Li^+^ demonstrates its potential application in the modified separator for Li‐S batteries. From the surface SEM (**Figure** **1**a–c) of the three prepared modified separators, the large pores of the unmodified separators are filled.^[^ ^29^ ^]^ The modified layer helps to barrier the pass‐through of polysulfides. Besides, the surface of the modified separators has uniform small pores, which is conducive to the absorption of electrolyte and the transportation of lithium ions. From the section images of the LTOPD and SPD (Figure 1d,e), it can be seen that the thickness of LTO and Super P modified layers is about 7.8 and 16.0 µm, respectively. The thickness of SPLTOPD is the addition of those of LTO and Super P modified layers (Figure 1f).

**Figure 2:**
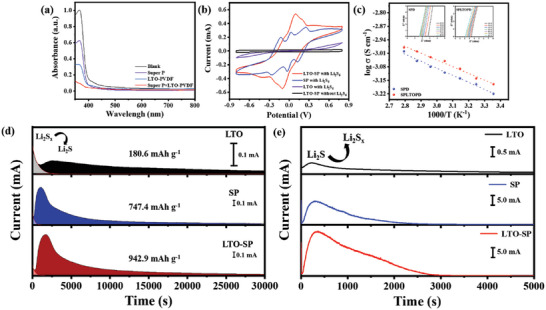
Effects of different modified materials on polysulfides and ionic conductivity test of the modified separators. a) UV‐visible absorption spectrums of Li_2_S_6_ solution adsorbed by different modified materials. b) CV curves of symmetrical cells with Li_2_S_6_ electrolyte at 5 mV s^−1^. c) The Arrhenius plots of different separators (insets: Corresponding EIS plots). d) Potentiostatic discharge profiles on different modified materials (at 2.08 V). e) Potentiostatic charge profiles for Li_2_S dissolution kinetics evaluation (at 2.40 V).

The adsorption ability of different materials on polysulfides is evaluated by the color change of the Li_2_S_6_ solution in addition of the modified materials (Super P, LTO‐PVDF, and Super P+LTO‐PVDF) with the same quality. The Li_2_S_6_ solution with Super P+LTO‐PVDF becomes clear and transparent after 12 h (Figure S3, Supporting Information), indicating this has excellent adsorption on polysulfides. The solution with LTO‐PVDF is clearer than that with Super P, indicating that LTO‐PVDF has stronger adsorption on polysulfides than Super P. Different modified materials were placed in Li_2_S_6_ solution for 12 h (**Figure** **2**a) to test the absorption ability of polysulfides from the UV‐visible absorption spectra. It could be seen that the absorbance of Super P+LTO‐PVDF@Li_2_S_6_ dispersion was the weakest compared with the other dispersions, which further proved that Super P+LTO‐PVDF could well adsorb polysulfides.^[^ ^40^ ^]^ The CV curves of symmetrical cells with different modified materials are used to judge their catalytic conversion ability on polysulfides. The symmetrical electrodes were prepared by coating different modified materials on aluminum foils followed by drying and cutting. When there is no Li_2_S_6_ in the electrolyte, there is no response current (Figure 2b), which shows that the response current is from the oxidation and reduction of Li_2_S_6_. The LTO symmetric cell has a slight response current, and the Super P symmetric cell has a significant response current, which may be related to the good conductivity of Super P. Relatively, the response current of the LTO‐SP symmetric cell is the largest, indicating that the LTO‐SP does have a good catalytic conversion ability on polysulfides.^[^ ^41^ ^]^

Stainless steel//stainless steel symmetrical cells with different separators were prepared. The ionic conductivity was obtained by electrochemical impedance spectroscopy (EIS), and the temperature range of the ionic conductivity test was 25–85 °C. There were short straight lines in the EIS plots (Figure 2c; Figure S4, Supporting Information), indicating that the carriers were ions.^[^ ^42^ ^]^ After calculation, the ionic conductivities of the LTOPD, SPD, and SPLTOPD are 0.42, 0.63, and 0.64 mS cm^−1^, respectively, at 25 °C. Compared with the ionic conductivity of the unmodified separator (DKJ‐14, 0.35 mS cm^−1^), ^[^ ^29^ ^]^ the ionic conductivity of the LTOPD is significantly improved at 25 °C, indicating that the LTO can promote the migration of Li^+^. Both Super P and LTO can promote the movement of ions. However, there is a limit for both of them. As a result, from the SPD to SPLTOPD, the increase of ionic conductivity is not much. From the test results of contact angle (Figure S5, Supporting Information), it can be seen that the contact angle is 17° when the electrolyte just drops on LTOPD, and changes to 10° after 5 s, indicating that the LTO can improve the affinity of the separator to the electrolyte. In addition, when the electrolyte just drops on the SPD and SPLTOPD, the contact angle is 11°, and changes to 6° and 5°, respectively, after 5 s, indicating that the SPLTOPD and electrolyte have better compatibility. The result is also consistent with the ionic conductivity results of different separators at 25 °C. The activation energies of ionic conduction for the LTOPD, SPD, and SPLTOPD are 12.2, 6.9, and 6.7 kJ mol^−1^, respectively. The comparison shows that the SPLTOPD has the lowest activation energy, indicating that the SPLTOPD has the lowest lithium ion transfer energy barrier.

The nucleation kinetics of Li_2_S were evaluated by potentiostatic discharge curves at 2.08 V (Figure 2d). According to Faraday's law, ^[^ ^43^ ^]^ the nucleation capacities of cells with different electrodes of LTO, SP, and LTO‐SP are 180.6, 747.4, and 942.9 mAh g^−1^, respectively. The comparison shows that the LTO‐SP provides the largest nucleation capacity, indicating that the LTO‐SP modified material is conducive to the precipitation of high‐capacity Li_2_S in Li‐S batteries. For the dissolution of Li_2_S (Figure 2e), the cell with the LTO‐SP electrode provided a higher current density than the two other cells, which is resulted from the better oxidation kinetics. These results further show that the LTO‐SP modified material can catalyze the conversion of polysulfides to Li_2_S and accelerate their redox kinetics in Li‐S batteries.

**Figure 3:**
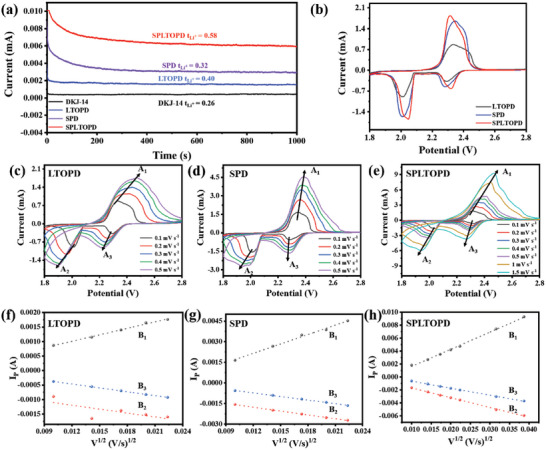
Li^+^ transference number and CV tests of the cells. a) *I*–*t* curves of lithium symmetrical cells with different separators at 10 mV. b) CV curves of the first circle of cells with different separators at 0.1 mV s^−1^. c–e) CV curves of cells with LTOPD, SPD, and SPLTOPD at various scan rates. f–h) the corresponding linear matching of peak point currents for the cells.

The Li^+^ transference numbers (t_Li+_) were measured by applying a constant voltage (10 mV) to the Li//Li symmetrical cells with different separators. Based on the calculation of i‐t curves and Nyquist plots (**Figure** **3**a; Figure S6, Supporting Information), the Li^+^ transference numbers of LTOPD, SPD, and SPLTOPD are 0.40, 0.32, and 0.58, respectively. Besides, the Li^+^ transference number of DKJ‐14 (0.26) was tested for comparison. The Li^+^ transference number of the separator through LTO modified is significantly increased, indicating that the LTO crystal is favorable for the mobility of Li^+^ cations.

The cells (S pieces as cathodes, lithium foils as anodes) were prepared with different separators and tested by cyclic voltammetry at 1.8–2.8 V. From the CV curves of the first cycle of cells with different separators at 0.1 mV s^−1^ (Figure 3b), it can be seen that there are two reduction peaks ≈2.3 and 2.0 V, ascribing to the reduction of low‐order sulfur to high‐order soluble lithium polysulfides (LiPSs) and further reduction to insoluble lithium sulfides (Li_2_S_2_/Li_2_S), respectively. The anodic peak ≈2.4 V correspond to the oxidation of sulfides.^[^ ^44^ ^]^ Comparison presents that the peak area of CV curve with the SPLTOPD is the largest, indicating that the SPLTOPD can promote redox kinetics, and improve the utilization of active sulphur. The distance between oxidation peak and reduction one is the narrowest, indicating that the SPLTOPD can reduce polarization in Li‐S batteries. From the CV curves of cells with different separators for the first three cycles at 0.1 mV s^−1^ (Figure S7, Supporting Information), it can be seen that the CV curves of the cell with the SPLTOPD have good coincidence, which further shows that the SPLTOPD can improve the utilization of sulfur. According to the CV curves of different cells with the prepared separators at various scan rates (Figure 3c–e), the calculated Li^+^ diffusion coefficients of the separators at different voltages are shown in Table S1 (Supporting Information). The Li^+^ diffusion coefficients of the SPLTOPD at each voltage are higher than those of the other two separators, which shows its promising application in Li‐S batteries. In addition, the cell with the SPLTOPD still has clear oxidation and reduction peaks even at 1.5 mV s^−1^, which is due to high mobility of Li^+^ cations.

**Figure 4:**
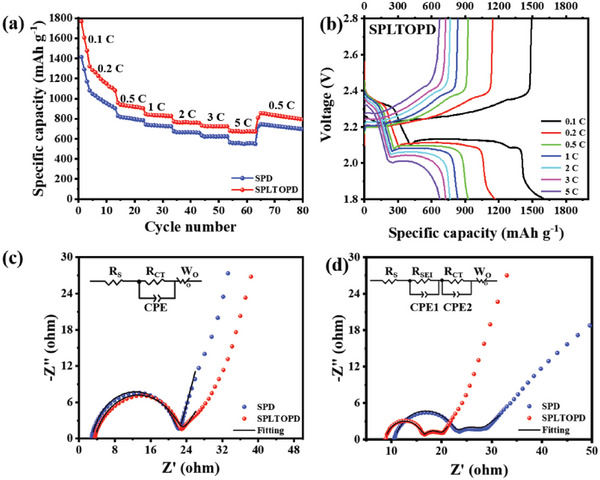
Rate performance and EIS tests. a) Rate performance and b) the corresponding charge/discharge profiles at various current densities of the Li‐S cell with the SPLTOPD. EIS plots and corresponding fittings c) before and d) after 100 cycles of cells with the SPD and the SPLTOPD (insets: equivalent circuit).

The rate performance of cells with different separators in **Figure** **4**a and Figure S8a (Supporting Information) shows that the cell with the SPLTOPD has excellent rate performance, and it delivers high capacities of 1606, 1168, 930, 834, 765, 726, and 674 mAh g^−1^ at current densities of 0.1, 0.2, 0.5, 1, 2, 3, and 5 C, respectively. In addition, from the charge and discharge curves under different current densities (Figure 4b; Figure S8b,c, Supporting Information), the cell with the SPLTOPD has two obvious reduction plateaus and a long oxidation one, indicating that the SPLTOPD can fully complete the redox reactions in Li‐S batteries. These results indicate the superiority of the SPLTOPD in Li‐S batteries.

EIS tests were carried out before and after 100 cycles with different separators (Figure 4c,d; Figure S8d, Supporting Information) to further study the reaction kinetics. Due to the dissolution and redistribution of the sulfur during the activation process, the resistance of the cells decreases sharply compared with that before cycling.^[^ ^45^ ^]^ The interfacial resistance (*R*_SEI_) of cells with the SPLTOPD is 7.7 Ω after 100 cycles, lower than those in the SPD (12.1 Ω) and the LTOPD (18.1 Ω) (Table S2, Supporting Information), indicating that a stable SEI film has been established for the Li‐S cells using SPLTOPD.^[^ ^46^ ^]^ The charge transfer resistances (*R*_CT_) of cells with the LTOPD, SPD, and SPLTOPD before cycling are 70.2, 19.3, and 19.1 Ω, respectively, and the R_CT_ after 100 cycles are 50.4, 4.1, and 3.2 Ω, respectively. The R_CT_ values of the cell with the SPLTOPD are the lowest, indicating that the SPLTOPD can improve the utilization of insulating sulfur species in Li‐S batteries. Besides, the *R*_CT_ values decreased significantly after coating of Super P, indicating that Super P can effectively reduce the charge transfer resistance of Li‐S batteries. The above results show that the SPLTOPD can effectively block the polysulfides and enhance the reaction kinetics of Li‐S batteries.^[^ ^47^ ^]^

**Figure 5:**
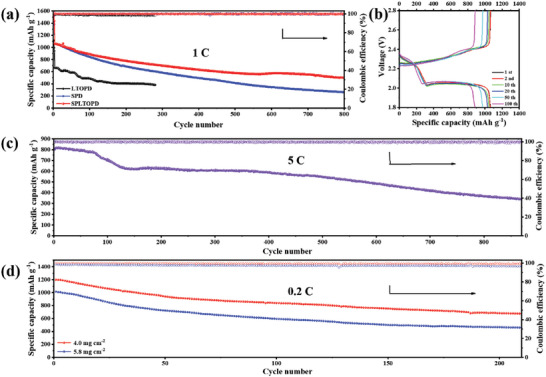
The cycling performance tests of Li‐S cells. a) Cycling performance at 1 C. b) Charge/discharge profiles with the SPLTOPD at 1 C. c) Cycling performance with the SPLTOPD at 5 C. d) Cycling performance of high areal sulfur‐loading (with the SPLTOPD, 0.2 C) after activation.

After the cathodes were fully activated after 3 cycles at 0.1 C, the cycling performance of the cells with the prepared separators at 1C was tested (**Figure** **5**a). The capacity of the cell with the SPD is only 262 mAh g^−1^ after 800 cycles, and its degradation is 0.095% per cycle. The cell with the LTOPD can cycle 280 times at 1 C with the initial capacity of only 666 mAh g^−1^, and the capacity degradation is 0.154% per cycle. In contrast, the initial capacity of the cell with the SPLTOPD is 1064 mAh g^−1^ and decreases to 497 mAh g^−1^ after 800 cycles at 1 C, with a degradation of 0.067% per cycle. This shows that the SPLTOPD can improve the utilization of sulfur and effectively suppress the ‘shuttle effect’ in Li‐S batteries. From the charge/discharge profiles at 1 C (Figure 5b; Figure S9, Supporting Information), it can be seen that the charging and discharging platforms of the cell with the SPLTOPD have no obvious deviation during the cycling, which further illustrates the superiority of the SPLTOPD in Li‐S batteries.

The long‐term cycling stability of Li‐S batteries at the high current density is also explored. Herein, the cell with the SPLTOPD was tested for ultra‐long cyclability at 5 C (Figure 5c). After activation, the initial specific capacity of the cell with the SPLTOPD is 808 mAh g^−1^, and it reaches the maximum specific capacity of 821 mAh g^−1^ after 9 cycles, because the cathode needs a certain activation process under high current density.^[^ ^48^ ^]^ After 870 cycles, the specific capacity can still be maintained at 341 mAh g^−1^, and the capacity degradation is only 0.066% per cycle with high coulomb efficiencies (> 98%) during the cycling. From the test results at the high current density, it can be seen that Li‐S batteries with the SPLTOPD show outstanding cycling stability.

High‐sulfur loading for S cathode is the key for Li‐S batteries to move from experimental stage to commercialization. When the sulfur loading is 4.0 mg cm^−2^, the maximum specific capacity of the cell with the SPLTOPD after activation could reach 1200 mAh g^−1^ at 0.2 C, and could retain 676 mAh g^−1^ after 210 cycles (coulomb efficiencies > 99%). When the sulfur loading increases to 5.8 mg cm^−2^, the maximum specific capacity after activation could reach 1016 mAh g^−1^ at 0.2 C, and could retain 460 mAh g^−1^ after 210 cycles (Figure 5d). Furthermore, after activation, the cell with the SPLTOPD still had the maximum capacity of 839 mAh g^−1^ at 0.2 C with sulfur loading of 7.6 mg cm^−2^ (Figure S10, Supporting Information) and maintains high coulomb efficiencies (> 96%) during the cycling. In addition, the electrochemical performance of the SPLTOPD was also compared with recent literature (Table S3, Supporting Information), and it can be seen that the SPLTOPD has high competitiveness in cycling performance.

Compared with the cathode before cycling (Figure S11a, Supporting Information), it can be seen that dense layers have been formed on the surface of the cathode after 100 cycles at 1 C for the cell with the base separator (DKJ‐14, Figure S11b, Supporting Information). This may be due to the deposition of insulating sulfur species on the cathode surface, ^[^ ^37^, ^49^ ^]^ which reduces the utilization rate of active substances. On the contrary, the cathode surface of the cell with the SPLTOPD has a porous structure and no dense layer after 100 cycles at 1C (Figure S11c, Supporting Information). The porous structure in the cathode is conducive to the deep diffusion of polysulfides inside the cathode.^[^ ^37^ ^]^ This result further demonstrates the superiority of the SPLTOPD in Li‐S batteries.

The H‐type cells were assembled to visually display the physical barrier of the modified separators on polysulfides (Figure S12, Supporting Information). When the Li_2_S_6_ solution and DME were injected into both sides of the cells, respectively, the original color of DME was maintained on the right side. After 2 h, the right side of the cell with the SPLTOPD remained clear and transparent. In contrast, some yellow polysulfides appeared on the right sides of cells with the SPD and LTOPD. This result shows that the SPLTOPD can effectively suppress the shuttle of polysulfides.

The symmetric Li//Li cell with the SPLTOPD was tested to show its effect on the lithium anodes, and the cell with original separator (DKJ‐14) was tested for comparison (Figure S13a, Supporting Information). After ≈500 h, the cell with the DKJ‐14 shows a sudden drop in voltage, which is caused by the short circuit due to the grown lithium dendrites penetrating the separator. In contrast, the cell with the SPLTOPD can cycle stably over 1800 h, indicating that the SPLTOPD can stabilize lithium deposition, because the LTO can homogenize the interface and prevent the formation of lithium dendrites.^[^ ^33^ ^]^

To visually observed the effect of the SPLTOPD on anodes, the lithium anodes of Li‐S cells after 100 cycles at 1 C with different modified separators were analyzed. From the SEM images of the anode surfaces (Figure S13b–d, Supporting Information), it can be seen that there are still some dendritic lithium and honeycomb corrosion pores on the lithium anode with the SPD. Since LTO can make Li^+^ cations deposit evenly, this phenomenon is obviously improved. As expected, the lithium anode of the cell with the SPLTOPD only presents the stable deposition of granular lithium after cycling. From the corresponding sectional SEM images of the anodes, it can be seen that the cell with the SPLTOPD has no corrosion layer on the corresponding sectional SEM images of the lithium anodes after cycling (Figures S13g and S14, Supporting Information). The above results show that the SPLTOPD can effectively protect the lithium anode and is conducive to the construction of Li‐S batteries with high safety performance. Furthermore, to prove the mobility of Li^+^ cations in the LTO, the lithium‐deposited morphologies in Cu foils of Li//Cu cells with the DKJ‐14 and LTOPD were tested (Figure S15, Supporting Information). Compared to the disorderly deposition of DKJ‐14, the LTOPD attained densely packed lithium deposition laid with regular shapes on the copper collector.

**Scheme 1:**
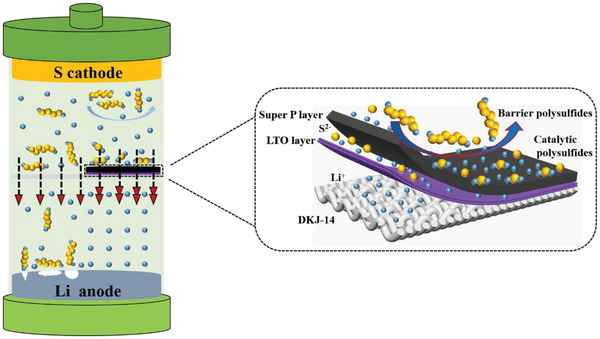
Schematic diagrams of actions of SPLTOPD and commercial separators (DKJ‐14) in the Li‐S battery.

The actions of the SPLTOPD and DKJ‐14 in the Li‐S battery are schematically shown in **Scheme** **1**, and it can be seen the superiority of the prepared separator. It is known that LTO is a commercial anode product for lithium‐ion batteries and can endure 10 C rate. It can form a stable barrier layer on the commercial separator to barrier the pass‐through of polysulfides. In addition, LTO is conducive to the transport of Li^+^, and barriers S^2−^. Super P can reduce charge transfer resistance and absorb polysulfides, and promote the conversion of soluble polysulfides to insoluble sulfides (Li_2_S_2_/Li_2_S) because of its good electronic conductivity, which accelerate the redox reaction in the Li‐S battery. Meanwhile, the SPLTOPD can prevent the aggregation of insulating sulfur species on the surface of the cathode and improve the utilization of active substances in the cathode. As a result, the Li‐S battery with the SPLTOPD exhibits good electrochemical performance under high sulfur loading and high current density, and the lithium anode has no lithium dendrites or a corrosion layer after 100 cycles at 1 C.

## 3 Conclusions

In summary, we prepared a separator modified with commercial LTO (Li_4_Ti_5_O_12_) and Super P for Li‐S batteries through a simple doctor coating method. LTO can improve the Li^+^ cation transport ability of the separators. Super P can reduce the charge transfer resistance of the Li‐S batteries and increase the kinetics of S/S^2−^ couple. The modified layer can effectively barrier the pass‐through of polysulfides, catalyze the reactions of polysulfides into S^2−^, and prevent the aggregation of insulating sulfur species on the surface of the cathode. As a result, the prepared Li‐S batteries can cycle 870 cycles at 5 C with small capacity attenuation of 0.066% per cycle. When the sulfur loading is 4.0 mg cm^−2^, the specific discharge capacity after activation at 0.2 C can reach 1200 mAh g^−1^, and when the sulfur loading is high up to 7.6 mg cm^−2^, it can still reach 839 mAh g^−1^. In addition, there are no lithium dendrites or a corrosion layer on the lithium anode of the cell with the SPLTOPD after 100 cycles at 1 C. It is no doubt that this work can greatly promote the commercialization of the functionally modified separators of Li‐S batteries.

## 4 Experimental Section

### Synthesis of Modified Separators

LTO (commercial anode product from Guoxuan, no carbon coating) and super P modified separators (SPLTOPD) were prepared by a simple doctor blading coating. First, LTO and PVDF with a mass ratio of 4:1 were put into a ball milling tank and milled overnight at 400 r min^−1^ to obtain LTO‐PVDF, and LTO‐PVDF (1 g) was dissolved in DMF, stirred for 6 h, and uniformly coated on the commercial separators (DKJ‐14). After drying overnight under vacuum at 60 °C LTO modified separators (LTOPD) were obtained. After PVDF was added to a certain amount of NMP and stirred until completely dissolved, then a certain amount of super P was added. After stirring for 6 h, it was coated on the LTOPD to obtain SPLTOPD. The loading amount of LTO and Super P on the SPLTOPD was about 2.64 mg cm^−2^. Super P modified separators (SPD) without LTO were made in the same way for comparison. All prepared separators were cut into 19 mm discs before use.

### Synthesis of S Cathodes

The preparation method of normal S cathodes was the same as we mentioned earlier.^[^ ^29^ ^]^ Briefly, a certain amount of CNTs was acidified in dilute nitric acid, washed and filtered until it was neutral, and dried in an 80 °C oven overnight. Sulfur and acidified CNTs with a mass ratio of 7:3 was put into the ethanol, stirred until the sulfur was completely dissolved. Then, deionized water was added until the sulfur was completely dissipated. The dispersion was washed, filtered, and vacuum dried overnight at 60 °C to obtain S composites. S composites, carbon black, and PVDF (mass ratio: 8:1:1) were put into NMP and stirred for 6 h until a uniform slurry was formed. The slurry was coated on aluminum foil, vacuum dried at 60 °C overnight, and cut into small discs to prepare S cathodes. The sulfur loading is ≈1.0 mg cm^−2^.

High sulfur‐loaded (≥4.0 mg cm^−2^) cathodes were prepared according to the previous report.^[^ ^26^ ^]^ Typically, CMC (80 mg) was added to deionized water (2.5 mL) and stirred until completely dissolved. Then, Super P (80 mg) was added, stirred until a uniform suspension was formed, and then the above S composite (560 mg) was added to obtain a uniform slurry. The high sulfur‐loaded cathodes were obtained by coating (carbon‐coated Al foil), vacuum drying, and cutting.

### Materials Characterization

The morphology of the samples was observed by a scanning electron microscope (Phenom ProX, SEM). The crystalline phase of the materials was tested by X‐ray diffraction analysis (Smart Lab3KW, XRD). A contact angle meter (Kino) was used to record contact angles. The Li_2_S_6_ solution adsorbed by the modified material was tested by a UV and visible spectrophotometer (UV‐2600).

### Synthesis of Coin Cells and Electrochemical Measurements

In a glove box filled with argon (O_2_<0.1 ppm, H_2_O<0.1 ppm), the cathodes, separators, electrolyte and anodes were successively encapsulated in the coin shells under the pressure of 500 kg cm^−2^ to prepare the CR2025 type coin cells. The amount of electrolyte in all cells is the same. The cycling and rate performance of the batteries were tested by the battery test system (LAND CT2001A, Wuhan, China). Cyclic voltammetry (CV, 1.8–2.8 V) and electrochemical impedance spectra (EIS, 10^−2^ ‐ 10^5^ Hz) were tested by electrochemical workstation (Chenhua, CHI760e).

The stainless steel sheet, separator, and stainless steel sheet were encapsulated in the battery shell (electrolyte was added to both sides of the separator), the ionic conductivity, and activation energy were calculated based on the EIS data of the battery at different temperatures.

The electrodes of Li_2_S_6_ symmetric cells were obtained by loading LTO and Super P on the aluminum foil. The electrolyte is 0.2 m Li_2_S_6_ solution in the electrolyte (1 m LiTFSI in DOL/DME = 1/1 by volume). The symmetric cells were assembled with the above electrodes as working and counter electrodes with 50 µL of Li_2_S_6_ solution. CV tests were carried upon a voltage window from −0.8 to 0.8 V.

Li_2_S_6_ (7 mL) solution and DME were separately placed on both sides of the H‐type battery, and different separators were placed in the middle.

## Conflict of Interest

The authors declare no conflict of interest.

## Supporting information

Supporting Information

## Data Availability

The data that support the findings of this study are available on request from the corresponding author. The data are not publicly available due to privacy or ethical restrictions.
