# Supplementary material for: A Separator with Double Coatings of Li4Ti5O12 and Conductive Carbon for Li‐S Battery of Good Electrochemical Performance
Source: Adv Sci (Weinh). 2023 May 18;10(22):2301386. doi: 10.1002/advs.202301386 (PMC10401180; doi:10.1002/advs.202301386)
Supplement: Supplementary file 1 — Supporting Information [file ADVS-10-2301386-s001.pdf]

ADVANCED SCIENCE
Open Access

Supporting Information

for *Adv. Sci.*, DOI 10.1002/advs.202301386

A Separator with Double Coatings of $Li_4Ti_5O_{12}$ and Conductive Carbon for Li-S Battery of Good Electrochemical Performance

Shuang Xia, Jie Song, Qi Zhou, Lili Liu, Jilei Ye, Tao Wang, Yuhui Chen, Yankai Liu, Yuping Wu* and Teunis van Ree*

Supporting Information: SI

A separator with double coatings of $Li_4Ti_5O_{12}$ and conductive carbon for Li-S battery of good electrochemical performance

Shuang Xia [a], Jie Song [a], Qi Zhou [a], Lili Liu [a*], Jilei Ye [a], Tao Wang [b], Yuhui Chen [a], Yankai Liu [c], Yuping Wu [a,b,*] , Teunis van Ree [d]

[a] State Key Laboratory of Materials-oriented Chemical Engineering & School of Energy Science and Engineering, Nanjing Tech University, Nanjing, 211816, China

[b] School of Energy and Environment, South East University, Nanjing, Jiangsu, 211189, China

[c] Hunan Bolt Power New Energy Co. Ltd, Dianjiangjun Industrial Park, Louxing District, Loudi 417000, Hunan Road, China

[d] Department of Chemistry, University of Venda, Thohoyandou 0950, South Africa

Materials

All of the chemicals mentioned in the synthesis steps were used directly without further treatment. Unmodified separators (DKJ-14) were purchased from Zhejiang DiKunJian New Energy Tech Co., Ltd. LTO was purchased from Hefei GuoXuan high tech power energy Co., Ltd. Super P was purchased from Tmall. Ltd. Electrolyte (LS-009) was purchased from DodoChem Co., Ltd. The anodes (lithium foils) were purchased from Tianjin Zhongneng Lithium Industry Co., Ltd. Bistrifluoromethane-sulfonimide lithium salt (LITFSI), dioxolane (DOL), carbon black (CB), 1,2-dimethoxyethane (DME), polyvinylidene difluoride (PVDF), *N*-methyl pyrrolidone (NMP), carbon nanotubes (CNTs), sublimated sulfur (S), lithium sulfide (Li_2S), and carboxymethyl cellulose (CMC) were purchased from Shanghai Aladdin Biochemical Technology Co., Ltd. The carbon-coated Al foil and carbon papers were purchased from Guangdong Canrd New Energy Technology Co., Ltd.

Preparation of Li_2S_6 solution and electrolyte

According to the following reaction equation (1):

$$Li_2S + 5S \rightarrow Li_2S_6 \qquad (S1)$$

Li_2S (0.92 g) and S (3.2 g) were dissolved in DME (100 ml) to obtain the Li_2S_6 solution (0.2 M).

S (640 mg) and Li_2S (184 mg) were dissolved in LS-009 (20 ml) to obtain the Li_2S_6 electrolyte (0.2 M).

Preparation of modified material pieces

According to our previous report, [1] some LTO-PVDF were placed in DMF, stirred for 6 h, and then uniformly coated on the aluminum foil. LTO pieces were obtained after vacuum drying at 60 ℃ overnight. A certain amount of Super P and PVDF were put into NMP, stirred evenly, coated evenly on LTO pieces, and vacuum dried at 60 ℃ to obtain LTO-SP pieces. Super P (SP) pieces were prepared in the same way for comparison. All prepared pieces were cut into 15 mm round pieces before use.

Preparation of Li_2S_8 electrolyte

The preparation of Li_2S_8 electrolyte was similar to that reported previously. [2] A blank electrolyte was prepared by dissolving 1 M LITFSI in DOL and DME (volume ratio was 1:1).

According to the following reaction equation (2):

$$Li_2S + 7S \rightarrow Li_2S_8 \quad \text{(S2)}$$

The sublimated sulfur (448 mg) and Li_2S (92 mg) were placed in the prepared blank electrolyte (10 mL) and stirred overnight at 50 ℃ to obtain the Li_2S_8 electrolyte (0.2 M).

The above polysulfides electrolytes were only used in some specific tests (catalytic performance tests, and Li_2S nucleation and dissolution tests), and the commercial electrolytes (LS-009) were used in other electrochemical tests.

Li_2S nucleation and dissolution test

According to our previous report, [2] the carbon papers were cut into small discs as fluid collectors, and the modified material was evenly coated on the carbon papers,

the area load on the discs was about 3.0 mg cm^{-2}, and the cathodes were obtained after drying. The separator was DKJ-14, and the anode was a lithium foil, the Li_2S_8 electrolyte (25 μL) and corresponding blank electrolyte (25 μL) were added on both sides of the separator respectively, and the cell was obtained by pressing them into the CR 2032 battery shell in the glove box filled with argon.

The Li_2S nucleation test was completed by discharging the above cell with a current of 0.134 mA to 2.09 V and keeping the voltage at 2.08 V.

The Li_2S dissolution test was completed by discharging the above cell at a current of 0.134 mA to 1.8 V, then converting Li_2S into soluble lithium polysulfides (LiPSs) at 2.4 V. [3]

Calculation

According to equation (3), the ionic conductivity of different separators at different temperatures can be calculated from the EIS.

$$\sigma = L / (R_b \times A) \tag{S3}$$

The symbols in the equation are ionic conductivity (σ), the thickness of separators (L), bulk resistance (R_b), and the contact area between the stainless steel sheet and the separator (A).

The activation energy of different separators can be calculated according to the Arrhenius equation (4).

$$\sigma = A \exp(-E_a / RT) \tag{S4}$$

The symbols in the equation are the pre-exponential factor (A), activation energy

(E_a), and the perfect gas constant (R).

The Li^+ transference number is calculated by equation (5).

$$t = I_S (V_1 - I_0 R_0) / I_0 (V_1 - I_S R_S) \quad (S5)$$

In the equation, I_0 and I_P are the initial current and steady-state current in the current curve respectively, V_1 is the transition potential (10 mV), R_0 and R_S are the AC impedance of the cell before and after polarization, respectively.

According to the Randles Sevcik equation (6), the Li^+ diffusion coefficients of cells with different separators can be calculated.

$$I_P = 2.69\times10^5 n^{1.5} A D_{Li+}^{0.5} C_{Li} V^{0.5} \quad (S6)$$

The symbols in the equation are the peak current (Ip), the number of electron transfers during the deintercalation of lithium (n), the contact area between active substance and electrolyte (A), the Li^+ diffusion coefficient (D_{Li+}), the concentration of Li^+ in an electrolyte (C_{Li}), and the scanning speed (V).

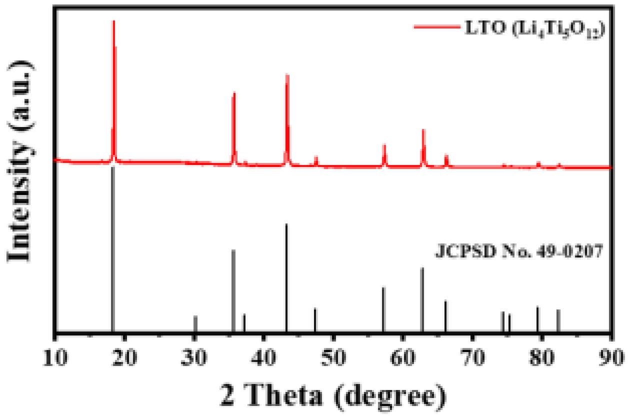

Fig. S1. The XRD of LTO.

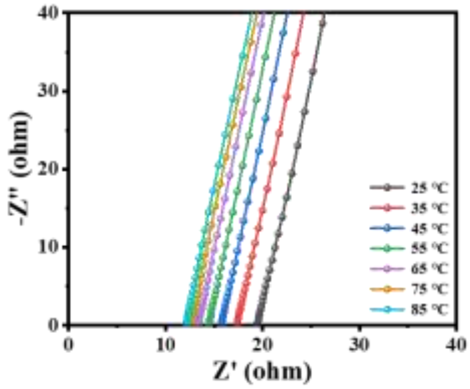

Figure S2. EIS plot of pure LTO pellet under different temperature.

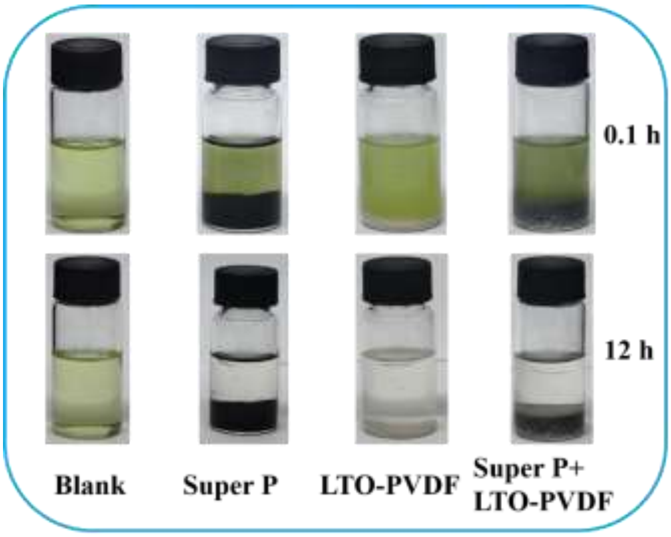

Fig. S3. Photographs of color changes of different modified materials in the Li_2S_6 solution.

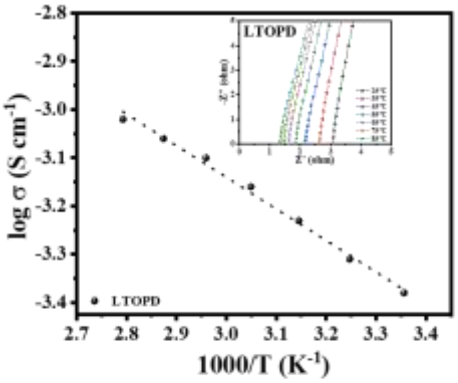

Fig. S4. The Arrhenius plots of LTPPD (insets: Corresponding EIS plots).

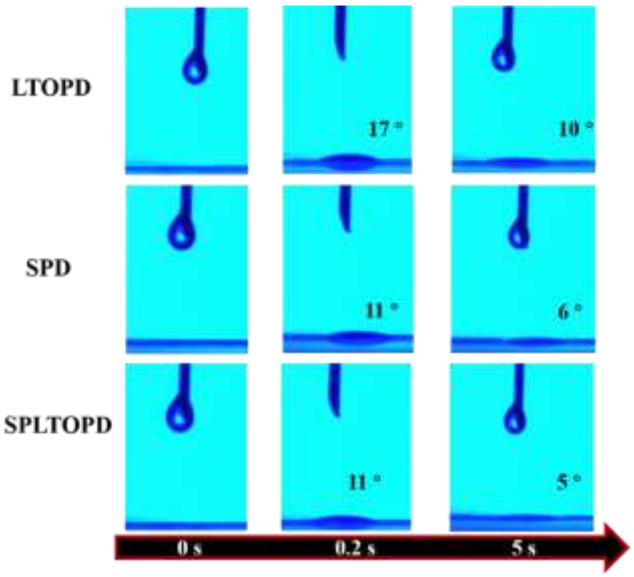

Fig. S5. Contact angle test for different separators.

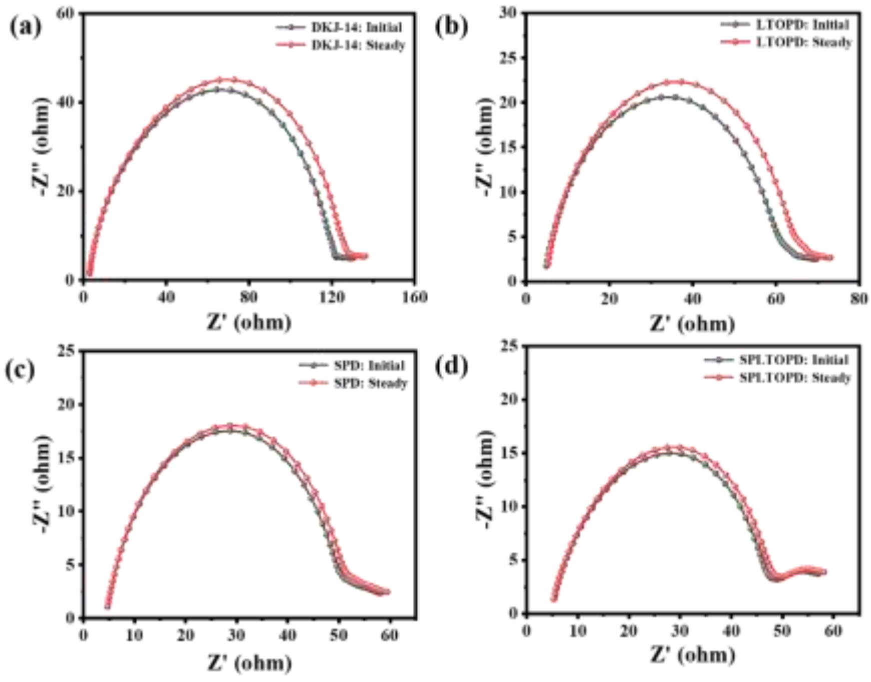

Fig. S6. Nyquist plots of symmetrical cells with different separators before and after polarization.

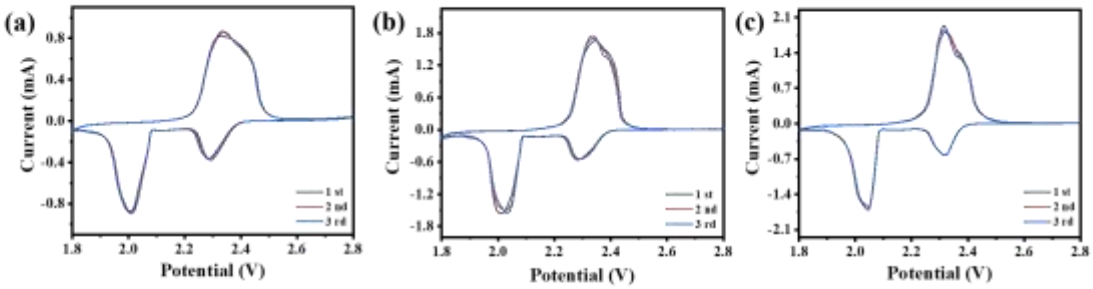

Fig. S7. (a), (b), and (c) CV Curves of cells with LTOPD, SPD, and SPLTOPD for the first three cycles at 0.1 mV s^{-1}.

Table S1. Li^+ diffusion coefficients of different separators.

Separators	D_{Li}^+(cm^2/s)-anodic peak around 2.5 V	D_{Li}^+(cm^2/s)-cathodic peak around 2.0 V	D_{Li}^+(cm^2/s)- cathodic peak around 2.3 V
LTOPD	5.9×10^{-9}	2.1×10^{-9}	2.1×10^{-9}
SPD	5.5×10^{-8}	9.3×10^{-9}	8.2×10^{-9}
SPLTOPD	7.5×10^{-8}	2.4×10^{-8}	1.2×10^{-8}

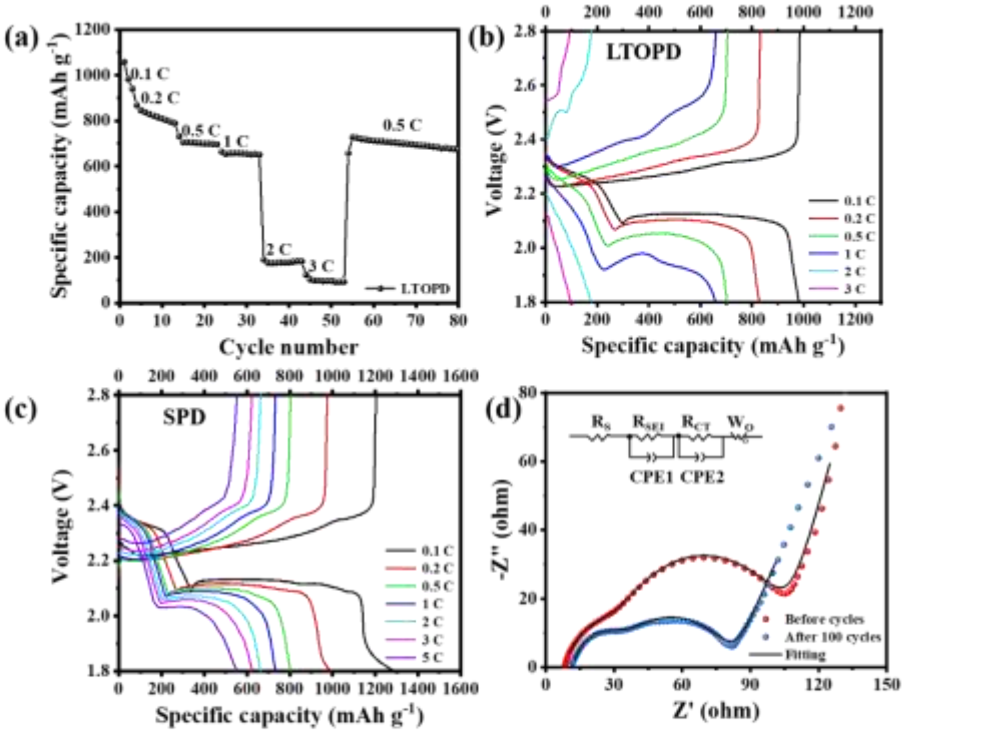

Fig. S8. (a) The rate performance of the cell with the LTOPD. (b)~(c) the charge/discharge

profiles at various current densities of the Li-S cells with the LTOPD and the SPD. (d) EIS plots and corresponding fittings before and after 100 cycles of Li-S cells with the LTOPD (insets: equivalent circuit).

Table S2. EIS parameters of the equivalent circuit simulation for the Li-S cells.

Cycle number	Resistance (Ω)	SPD	SPLTOPD	LTOPD
Before cycling	R_S	2.8	3.5	8.0
	R_{SEI}	-	-	22.3
	R_{CT}	19.3	19.1	70.2
100 cycles	R_S	10.6	8.7	11.5
	R_{SEI}	12.1	7.7	18.1
	R_{CT}	4.1	3.2	50.4

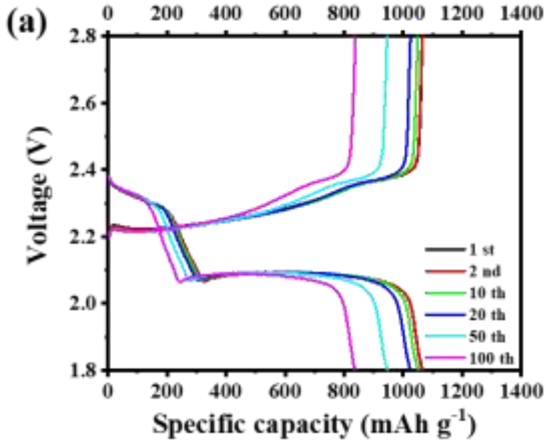

Fig. S9. Charge/discharge profiles of Li-S cells with the (a) the SPD and (b) the LTOPD at 1 C.

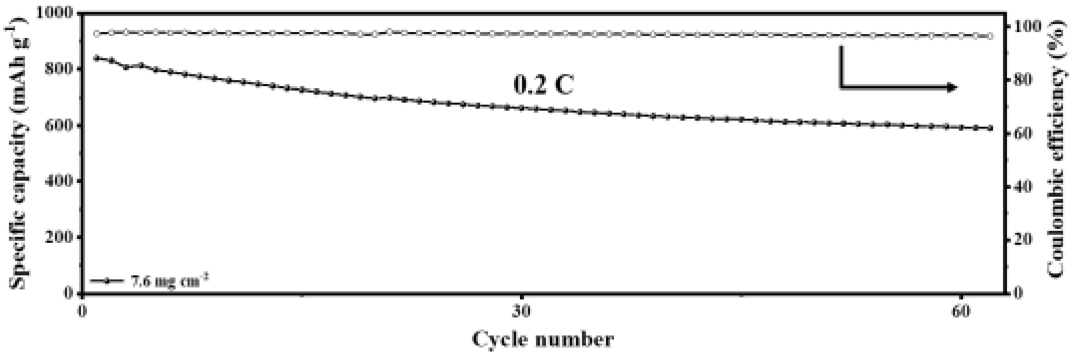

Fig. S10. Cycling performance of Li-S batteries after activation of the cell with the SPLTOPD at a sulfur load of 7.6 mg cm^{-2} (0.2 C).

Table S3. A comparison of electrochemical performance with the SPLTOPD and other recent reports.

**Cathode**	**Separator**	**S loading (mg cm^{-2})**	**Initial capacity (mAh g^{-1})**	**Cycle number**	**Decay rate per cycle (%)**	**Ref.**
**S/CNT**	**SPLTOPD**	**~1.0**	**1064**<br>**808**	**800 (1 C)**<br>**870 (5 C)**	**0.067**<br>**0.066**	**This work**
S/CNT	C@COF	1.2-1.5	1013.5	500 (1 C)	0.07	1[2]
S/GO	Asy-PP/Li-Mg	1.0-1.3	1116	400 (1 C)	0.07	2[4]
S/C	MoS_2	NA	808	600 (0.5 C)	0.083	3[5]
S/KB	MWCNTs/NCQDs/PP	1.3-1.5	1330.8	500 (0.5 C)	0.1	4[6]
S/CB	SCOF-2	1.5	795	800 (1C)	0.05	5[7]

S/C	MoS_2@NC	1.2	1020	500 (0.5 C)	0.0874	6[8]
S/C	SrF_2-G/PP	NA	NA	300 (0.5 C)	0.07	7[9]
S/C	CNMCO/PP	NA	1029	400 (0.5 C)	0.096	8[10]
S/CB	Janus nanofabric	1.5	890	300 (0.5 A g^{-1})	0.117	9[11]
S/super P	PyBBT-COF	1.0	1249	100 (0.2 C)	0.27	10[12]
S/MWCNT	CNF/Co-Co_9S_8-NC	1.5	746	300(2 C)	0.083	11[13]
CNT/S	Fe_3C-FeN@NCF/PP	~1.0	895	800(1 C)	0.07	12[14]
S/C	VN_{1-x}@V-NC@PP	1.5	NA	500 (2 C)	0.071	13[15]

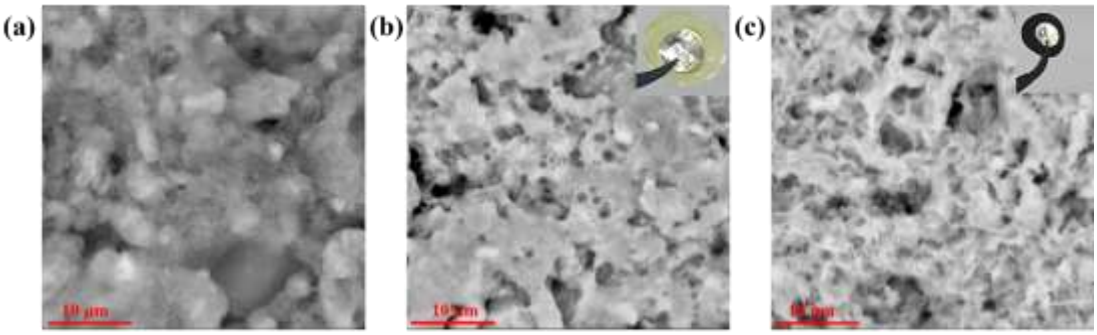

Figure S11. SEM of cathodes. (a) before cycling. SEM of the Li-S cells with the (b) DKJ-14 and (c) SPLTOPD after 100 cycles at 1 C (insets: photographs of the cathodes and separators).

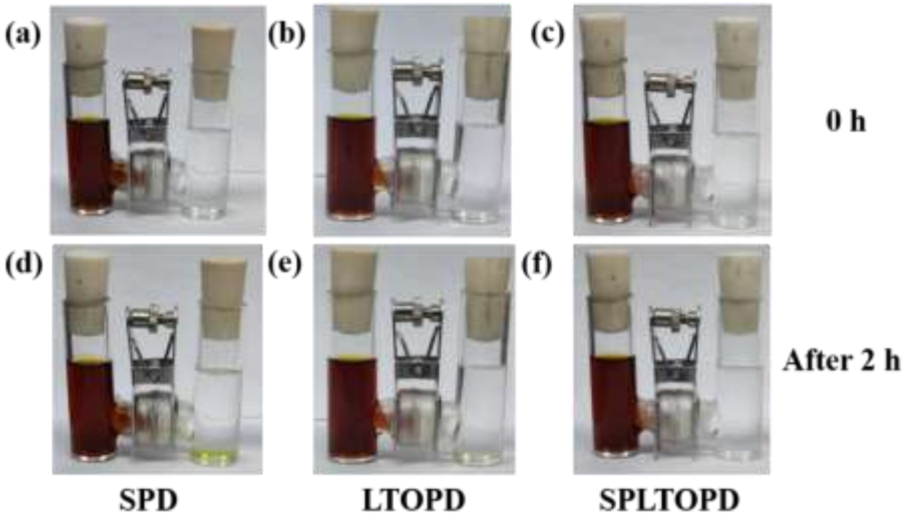

Fig. S12. Photographs of polysulfides diffusion test in H-type cells with (a, d) the SPD, (b, e) the LTOPD, and (c, f) the SPLTOPD.

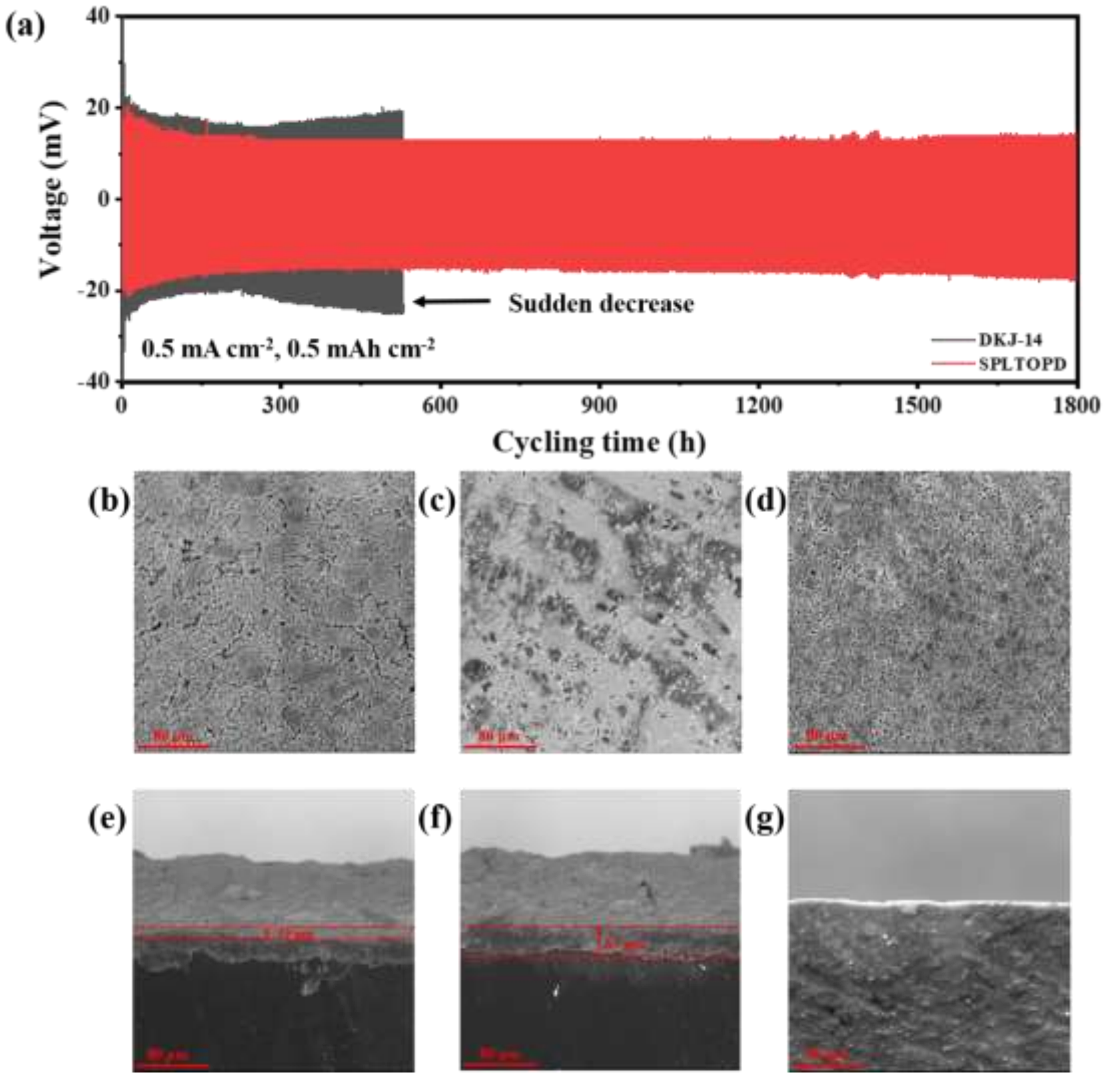

Fig. S13. Li stripping/plating behavior and the SEM of lithium anodes. (a) Voltage profiles of symmetric lithium cells with the SPLTOPD and DKJ-14 at 0.5 mA cm^{-2}. images of lithium anode of Li-S cells with the (b) LTOPD, (c) SPD, and (d) SPLTOPD after 100 cycles at 1 C, and (e~g) the corresponding cross-section.

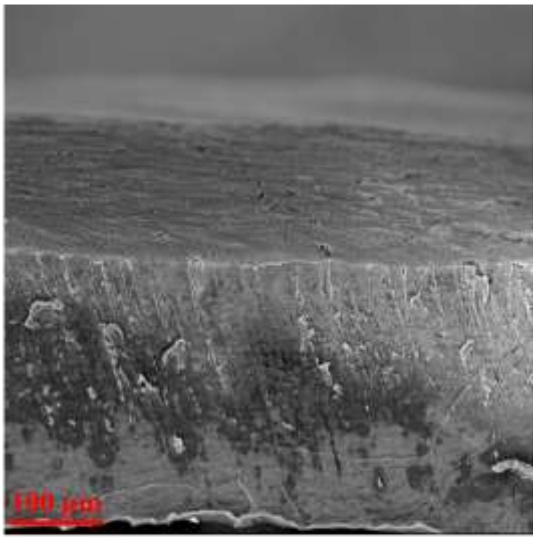

Fig. S14. The SEM image (anode) of the Li-S cell with the SPLTOPD for 100 cycles at 1 C (the other side of the cut).

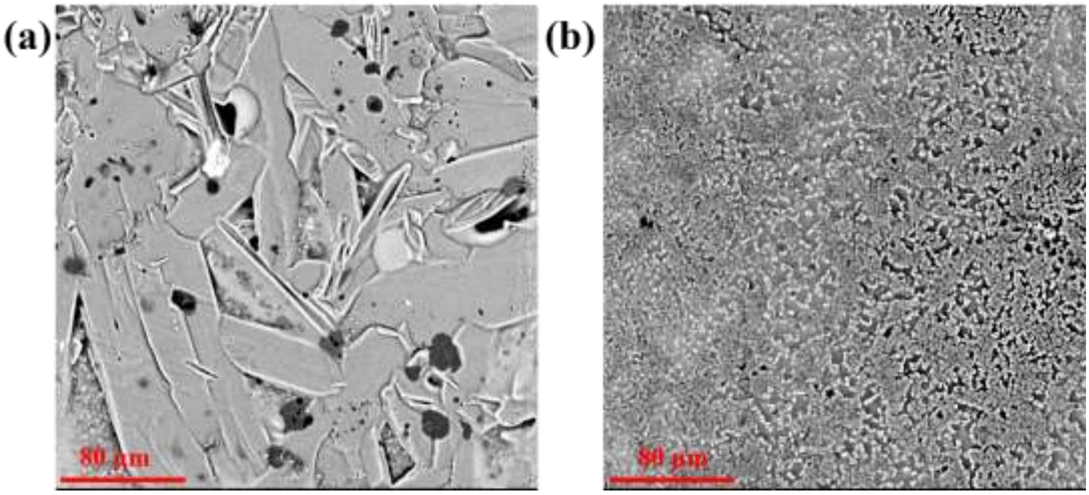

Figure S15. The lithium-deposited morphologies in Cu foils of Li//Cu cells with the (a) DKJ-14 and (b) LTOPD at 0.5 mA cm^{-2} (5 h).

References

1. S. Xia, W. Huang, W. Yan, X. Yuan, X. Chen, L. Liu, L. Fu, Y. Zhu, Q. Huang, Y. Wu, T. van Ree and R. Holze, *ChemElectroChem*, **2022**, *9*, 202200301.
2. W. Yan, X. Gao, J. L. Yang, X. Xiong, S. Xia, W. Huang, Y. Chen, L. Fu, Y. Zhu and Y. Wu, *Small*, **2022**, *18*, e2106679.
3. H. Yuan, H.-J. Peng, B.-Q. Li, J. Xie, L. Kong, M. Zhao, X. Chen, J.-Q. Huang and Q. Zhang, *Advanced Energy Materials*, **2019**, *9*, 1802768.
4. W. Yan, J. L. Yang, X. Xiong, L. Fu, Y. Chen, Z. Wang, Y. Zhu, J. W. Zhao, T. Wang and Y. Wu, *Advanced Science*, **2022**, *9*, 2202204.
5. Z. A. Ghazi, X. He, A. M. Khattak, N. A. Khan, B. Liang, A. Iqbal, J. Wang, H. Sin, L. Li and Z. Tang, *Adv Mater*, **2017**, *29*, 1606817.
6. Y. Pang, J. Wei, Y. Wang and Y. Xia, *Advanced Energy Materials*, **2018**, *8*, 1702288.
7. J. Xu, S. An, X. Song, Y. Cao, N. Wang, X. Qiu, Y. Zhang, J. Chen, X. Duan, J. Huang, W. Li and Y. Wang, *Adv Mater*, **2021**, *33*, e2105178.
8. J. Wu, X. Li, H. Zeng, Y. Xue, F. Chen, Z. Xue, Y. Ye and X. Xie, *Journal of Materials Chemistry A*, **2019**, *7*, 7897-7906.
9. W. Jing, J. Zu, K. Zou, X. Dai, Y. Song, J. Han, J. Sun, Q. Tan, Y. Chen and Y. Liu, *Journal of Materials Chemistry A*, **2022**, *10*, 4833-4844.
10. W. Sun, Y.-C. Lu and Y. Huang, *Journal of Materials Chemistry A*, **2021**, *9*, 21184-21196.
11. M. Chen, Z. Chen, X. Fu and W.-H. Zhong, *Journal of Materials Chemistry A*, **2020**, *8*, 7377-7389.
12. R. Wang, Q. Cai, Y. Zhu, Z. Mi, W. Weng, Y. Liu, J. Wan, J. Hu, C. Wang, D. Yang and J. Guo, *Chemistry of Materials*, **2021**, *33*, 3566-3574.
13. N. Gao, X. Shen, Y. Liu, Z. Xu, X. Wang, H. Liu, Y. Ren, S. Chen, and Z. Li, *Journal of Materials Chemistry A* **2023**, *11*, 5212-5221.
14. Mengdi Zhang, Jiawei Mu, Yanan Li, Yuanyuan Pan, Zhiliang Dong, Bei Chen, Shiwei Guo, Wenhan Yuan, Haiqiu Fang, Han Hu, and Mingbo Wu, *Journal of Energy Chemistry*. **2023**, *78*, 105-114.
15. Y. Zhang, C. Ma, C. Zhang, L. Ma, S. Zhang, Q. Huang, C. Liang, L. Chen, L. Zhou, and W. Wei, *Chemical Engineering Journal*. **2023**, *452*, 139410.
